# Depressive and Anxiety Disorders in Systemic Lupus Erythematosus Patients without Major Neuropsychiatric Manifestations

**DOI:** 10.1155/2016/2829018

**Published:** 2016-09-26

**Authors:** Ru Bai, Shuang Liu, Yueyin Zhao, Yuqi Cheng, Shu Li, Aiyun Lai, Zhongqi Xie, Xinyu Xu, Zhaoping Lu, Jian Xu

**Affiliations:** ^1^Department of Rheumatology and Immunology, First Affiliated Hospital of Kunming Medical University, Kunming 650032, China; ^2^Department of Psychiatry, First Affiliated Hospital of Kunming Medical University, Kunming 650032, China

## Abstract

Depressive and anxiety disorders are frequently observed in patients with Systemic Lupus Erythematosus (SLE). However, the underlying mechanisms are still unknown. We conducted this survey to understand the prevalence of depression and anxiety in SLE patients without major neuropsychiatric manifestations (non-NPSLE) and to explore the relationship between emotional disorders, symptoms, autoantibodies, disease activity, and treatments in SLE. 176 SLE patients were included, and SLE disease activity index (SLEDAI), Hamilton Depression Rating Scale (HAMD), and Hamilton Anxiety Rating Scale (HAMA) were recorded to evaluate their disease activity and emotional status. We found that depressive and anxiety disorders were common among SLE patients: 121 (68.8%) patients were in depression status while 14 (8.0%) patients could be diagnosed with depression. Accordingly, 101 (57.4%) were in anxiety status and 21 (11.9%) could be diagnosed with anxiety. Depression was associated with disease activity, and anxiety was associated with anti-P0 antibody, while both of them were associated with proteinuria. HAMA and HAMD scores were in strong positive correlation and they were independent risk factors of each other. We concluded that the high prevalence of depression and anxiety and the association between depression and SLE disease activity might reveal the covert damage of central nervous system in SLE. The role of anti-P0 antibody in SLE patients with emotional disorders warrants more researches.

## 1. Introduction

Systemic Lupus Erythematosus (SLE) is a typical connective tissue disease with multiple organs involved, including central nervous system (CNS), peripheral nervous system (PNS), and autonomic nervous system. Neuropsychiatric symptoms are common and serious manifestations and sometimes can cause disability or death. Major neurological and mental disorders like stroke or schizophrenia are not as common as subtle ones such as headaches, emotional disorders, and cognitive deficiencies. Clinical evaluations are the major diagnostic methods of neuropsychiatric Systemic Lupus Erythematosus (NPSLE), though it is often quite difficult to make a definite diagnosis, mostly only “hypothetical” ones [1]. Sometimes NPSLE can present as cognitive dysfunction and emotional disorders and affect patients' quality of life severely [2, 3]. Severe neuropsychiatric symptoms are reported to be associated with long-term progression of disease and could cause death in 7–19% cases [4]. Thus, it is important for clinical physicians to recognize signs and symptoms of NPSLE in early stage.

SLE is characterized by repeated flares and remissions of variable symptoms and signs, of which proteinuria, rashes, and arthritis are the most common ones. Besides those frustrating symptoms, social stress such as loss of working abilities, decreased incomings, and limitations in social activities are also a major problem. Altogether they may cause emotional disorders like depression and anxiety in SLE patients [5]. Some researchers believed that emotional disorders could be the initial symptoms of NPSLE as a result of inflammation and consequences of certain antibodies [6–8]. However, emotional disorders cannot always be recognized in early phase by clinicians due to lack of awareness [9]. In SLE patients without “obvious” or major neuropsychological symptoms like seizures or mental disorders, the so-called “non-NPSLE” patients, the incidence and characteristics of emotional disorders are not completely studied. Several studies in the potential neurobiological mechanisms indicated autoantibody production, microvasculopathy, and proinflammatory cytokines might play essential roles [4]. Thus, it is crucial to identify specific autoantibodies and tests to help recognize emotional disorders. Our study included 176 non-NPSLE patients with normal conventional brain imaging and no history of neuropsychiatric disease and intended to explore the prevalence of depression and anxiety in these patients and understand the relationship between emotional disorders, symptoms, autoantibodies, disease activity, and treatments in SLE.

## 2. Materials and Methods

### 2.1. Subjects

176 SLE patients were recruited from inpatient and outpatient centers from the Department of Rheumatology and Immunology of the First Affiliated Hospital of Kunming Medical University, Kunming, Yunnan, China, which is a member unit of Chinese SLE Treatment and Research Group (CSTAR). All patients were from Chinese Han population. All participants signed informed consents after a complete description of the study and experienced full physical examinations and neuropsychiatric scales to evaluate disease activity and neuropsychiatric status by a multidisciplinary team with rheumatologists, neurologists, and psychiatrists. The major scales included a self-made questionnaire, Hamilton Depression Rating Scale (HAMD), Hamilton Anxiety Rating Scale (HAMA), and Systemic Lupus Erythematosus Disease Activity Index (SLEDAI) to get the general conditions, emotional status, and disease activity of patients [10–12]. All patients had routine brain magnetic resonance imaging (MRI) scans to rule out major CNS diseases. Full set of autoantibodies including antinuclear antibody (ANA), anti-ribosomal P0 antibody (anti P0 antibody), anti-double stranded deoxyribonucleic acid (dsDNA) antibody, anti-Sm antibody, anti-U1-ribonucleoprotein (U1-RNP) antibody, anti-SSA-52 kD antibody, anti-SSA-60 kD antibody, anti-SSB antibody, anti-histones antibody, anti-nucleosome antibody, anti-cardiolipin (aCL) antibody, and lupus anticoagulant complex (LAC) was tested. This research was approved by the Institutional Review Board of Kunming Medical University, Yunnan Province, China (ClinicalTrials.gov: NCT00703742).

The exclusion criteria included the following: (1) patients with rheumatoid arthritis (RA), systematic sclerosis (SSc), idiopathic or secondary Sjogren's syndrome (SS) or other connective tissue diseases (CTD), or drug-induced SLE; (2) patients with serious disorders of heart, liver, kidney, or other major organs; (3) patients with disorders of central or peripheral nervous system; (4) patients with conditions which could induce cerebral atrophy such as stroke, kidney failure, high blood pressure, diabetes, and drug or alcohol dependence; (5) patients with a history of epilepsy, except for infantile febrile convulsion.

### 2.2. Statistical Analysis

Statistical analysis was conducted with SPSS 17.0 (SPSS Inc., 1989–2004). Variables were tested to find whether they met normal distribution. Normally distributed variables were shown with mean and standard deviation (SD), while nonnormally distributed ones were shown with median and interquartile range (IQR). Univariate comparisons between categorical variables were performed by chi-square test, while Mann-Whitney test was performed to evaluate numerical variables. For correlation between two numerical variables, we used Pearson's or Spearman's correlation. Finally, we used binary logistic regressions to find possible risk factors of depression and anxiety. The results were considered significant when *p* < 0.05.

## 3. Results

### 3.1. General Conditions of Patients

This study included 176 SLE patients with 23 males and 153 females. Their age ranged within 13–52 years with a mean age of 30.5. 92 (52.3%) patients were newly diagnosed. Patients treated with glucocorticoids (GCs), cyclophosphamide (CTX), and hydroxychloroquine (HCQ) were 131 (74.4%), 37 (21.0%), and 82 (46.6%), respectively. The results of general conditions were shown in Table 1.

### 3.2. Emotional Disorder Conditions

Depression was evaluated via HAMD scores, with score less than 7 as normal, 7–17 as mild or probable depression, 18–24 as moderate or definite depression, and more than 24 as severe depression. 121 (68.7%) patients got scores defined as mild to severe depression, 107 (60.8%) as mild depression, 13 (7.4%) as moderate depression, and 1 (0.6%) as severe depression. Anxiety was evaluated through HAMA scale, with score less than 7 as normal, 7–14 as mild or probable anxiety, 15–21 as moderate or definite anxiety, and more than 21 as severe anxiety. Anxiety was present in 101 (57.4%) patients while 80 (45.5%) patients had mild anxiety, 19 (10.8%) had moderate anxiety, and 2 (1.1%) had severe anxiety. Patients with SLEDAI scores less than 9 were inactive while those with scores of 9 and above had active disease status.

### 3.3. Association between Emotional Disorders and Clinical Phenotypes of SLE

**Figure 1:**
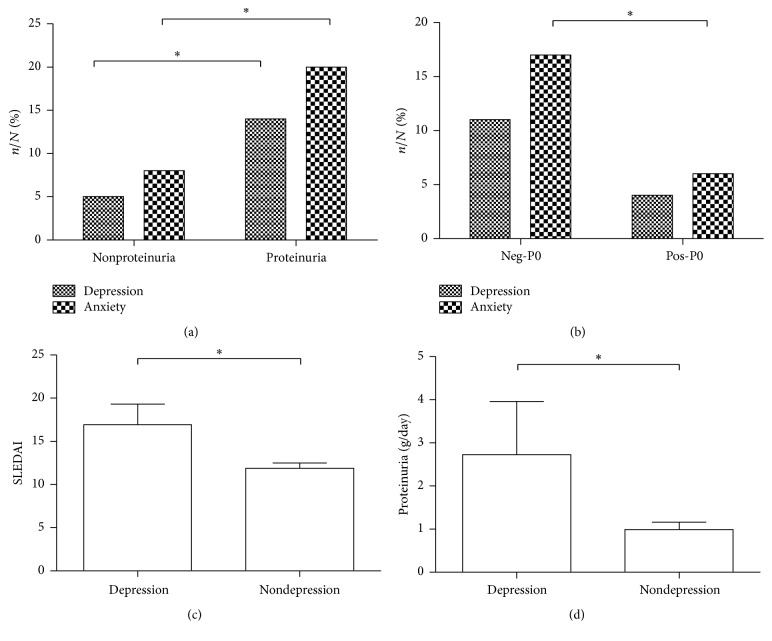
Association between emotional disorders and clinical phenotypes of SLE. (a) The prevalence of depression and anxiety was higher in patients with proteinuria; (b) the prevalence of anxiety was higher in patients with negative P0 antibody; (c) the SLEDAI score of depression patients was higher; (d) depression patients were with more proteinuria. *∗* showed *p* < 0.05.

We considered patients with HAMD scores more than 17 as definite depression and others as nondepression. Chi-square analysis showed the prevalence of depression was higher in patients with proteinuria, pyuria, hematuria, and anxiety (14.3% versus 4.8%, *χ* ^2^ = 4.142, *p* = 0.042; 13.8% versus 4.7%, *χ* ^2^ = 4.551, *p* = 0.033; 16.1% versus 4.3%, *χ* ^2^ = 5.502, *p* = 0.019; 42.9% versus 3.2%, *χ* ^2^ = 25.129, *p* = 0.000, resp.), while the prevalence of anxiety was higher in patients with elder age, alopecia, proteinuria, negative ant-P0 antibody, and depression (17.9% versus 6.7%, *χ* ^2^ = 5.127, *p* = 0.024; 24.4% versus 7.6%, *χ* ^2^ = 6.980, *p* = 0.008; 19.6% versus 7.7%, *χ* ^2^ = 4.968, *p* = 0.026; 16.7% versus 5.9%, *χ* ^2^ = 4.383, *p* = 0.036; 64.3% versus 7.4%, *χ* ^2^ = 25.129, *p* = 0.000, resp.) (see Figures 1(a) and 1(b)).

Mann-Whitney analysis showed that the depression group had a higher score of SLEDAI and HAMA and higher proteinuria (16.93 versus 11.89, *p* = 0.027; 16.64 versus 7.23, *p* = 0.000; 2.73 g/day versus 0.99 g/day, *p* = 0.010, resp.) (see Figures 1(c) and 1(d)).

**Figure 2:**
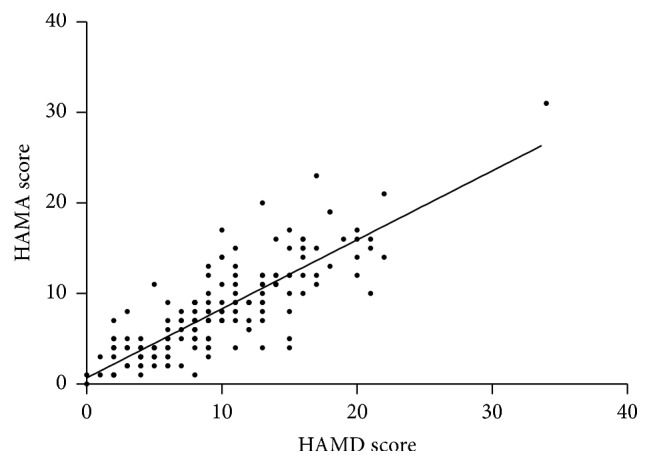
The correlation between HAMD and HAMA scores (*r* = 0.82, *p* = 0.000).

Spearman correlation tests showed that HAMA scores were in strong positive correlation with HAMD scores (*r* = 0.82, *p* = 0.000) (see Figure 2). The cumulative dosage of HCQ was in positive correlation with both HAMD and HAMA scores (*r* = 0.173, *p* = 0.038; *r* = 0.243, *p* = 0.003, resp.). The age and disease duration were also in positive correlation with HAMA scores (*r* = 0.182, *p* = 0.016; *r* = 0.264, *p* = 0.001, resp.).

However, when we analyzed the possible risk factors we got from the analysis above in binary logistic regression, we found that only pyuria, hematuria, and HAMA score were the risk factors of depression, and proteinuria, SLEDAI, and cumulative dosage of HCQ were not significantly relevant. As to anxiety, we found alopecia and HAMD score were the risk factors, and age had a trend, while disease duration, proteinuria, anti-P0 antibody, and cumulative dosage of HCQ were not significantly relevant with anxiety (see Tables 2(a) and 2(b)).

## 4. Discussion

Neuropsychiatric symptoms are major symptoms in SLE patients, and 19 of them are considered as NPSLE. NPSLE patients may have poorer prognosis and higher mortality [13]. Severe NPSLE like seizures, stroke, or mental disorders are well recognized in clinical situations. However, due to lack of awareness, subtle NPSLE syndromes like emotional disorders including depression and anxiety are not well recognized. Some doctors may even consider these symptoms as “non-NPSLE” when the patients have no history of “neuropsychiatric disorders” and normal conventional brain MRI scans, just like the patients we recruited. We can recognize patients with emotional disorders with thorough psychiatric evaluations in early phase. The prevalence of depression was reported to be 10.8–68%, while that of anxiety was 15.6–46.5% [6, 14–16]. Our study showed that, in the patients of our sample, depression and anxiety were quite common in non-NPSLE patients, with a proportion of 68.7% and 57.4%, respectively, which is consistent with prior studies.

The association between depressive and anxiety disorders and clinical symptoms was quite different in various studies. Factors like ethnicity, rashes, disease activity, and certain antibodies like anti-P0 antibody or aCL antibody were all involved [16–21]. In our study, we found that depression was associated with urinary symptoms and SLEDAI, and anxiety was associated with negative P0 and age, while proteinuria was associated with both of them and cumulative dosage of HCQ was in positive correlation with both of them. Depression and anxiety were the most important predictors of each other.

The relationship between disease activity and depression and anxiety was studied by several researchers before and the results were inconsistent. Julian et al., Nery et al., and Bachen et al. reported disease activity was relevant with depression and anxiety, while Huang et al. and Hanly et al. found no relevance in their studies [17–21]. Thus, Palagini et al. summarized that as lack of studies and methodological limitations; the relationship might remain contradictory before more studies [22]. Our study showed association between depression and disease activity. And both depression and anxiety were associated with proteinuria and higher cumulative dosage of HCQ, which might reveal higher disease activity. Thus, we assumed that emotional disorders were related to disease activity.

Anti-ribosomal P (RP) antibody targets C-terminal region of ribosomal P protein, mainly ribosomal phosphoprotein P0, P1, and P2. Anti-RP antibody was considered as a specific antibody of SLE and one of the most relevant antibodies of NPSLE [23–27]. The prevalence of anti-RP antibody in SLE patients ranged from 6% to 42% and was supposed to be higher in Asian patients due to ethnic differences [25]. Several studies found that anti-RP antibody was associated with psychosis and depression [24–27]. Karimifar et al. found that the association occurred in early course of SLE patients and believed that anti-RP antibody could cause certain NPSLE symptoms [24]. The detection of anti-RP antibody in cerebrospinal fluid (CSF) was considered to be more meaningful than that in serum [28, 29]. And some researchers injected anti-RP antibodies directly into the brain ventricles of mice to induce depression-like behaviors, which could get improved by antidepressant drugs and blocking the antibodies [30–32]. However there were some studies that showed no relevance between anti-RP antibodies and neuropsychiatric symptoms [19, 33, 34]. Iseme et al. believed that anti-RP antibody could upregulate proinflammatory cytokines like interferon and could cause neuronal death via apoptosis, which was the underlying mechanism of neuropsychiatric symptoms [35]. Arnett et al. found that anti-RP antibody was strongly influenced by certain MHC class II alleles which might suggest the underlying genetic mechanism [36].

As to anxiety, Aldar et al. found that anti-RP antibodies were higher in anxious childhood-onset SLE patients [37], while most other studies showed no relation between anti-RP antibody and anxiety [6, 25, 38–40]. In our study, we found no association between anti-P0 antibody and depression. However, we found that patients with anti-P0 antibody had a lower chance of anxiety. The controversial results might be due to the heterogeneity of the disease. No association between anti-P0 antibody and anxiety was found in binary logistic regression; thus, this result requires further confirmation. Although the relationship between anti-RP antibody and NPSLE, especially depression, was quite certain, the variety of symptoms and classifications of NPSLE made it hard for us to understand the relationship between anti-RP antibody and specific NPSLE manifestations like anxiety. Whether it was significant needed more statistics. Another possible explanation might be that we chose “non-NPSLE” patients in this study, in which the proportion of positive anti-P0 antibody was low.

NPSLE could be the original manifestation of SLE. Depression and anxiety are major emotional disorders in SLE patients. However, lack of awareness and difficulties in early recognition make it hard to get early treatment for patients suffering from these conditions. Our study found that depression and anxiety were really common in SLE patients considered as “non-NPSLE,” and they were strong risk factors of each other. Depression was associated with disease activity, while both depression and anxiety were associated with proteinuria and higher cumulative dosage of HCQ, which might reveal the higher disease activity. This might suggest that emotional disorders could be early phase of SLE brain damage. Unexpectedly, anxiety was associated with negative anti-P0 antibody, which should be reexamined by more studies to find out the role of anti-P0 antibody in depression and anxiety.

## Figures and Tables

**Table 1:** General conditions of 176 SLE patients.

	Median, IQR
Age (year)	29.5 (24, 37)
Disease duration (month)	8 (1, 32)
Proteinuria (g/day)	0.295 (0.1, 1)
SLEDAI	11 (6, 17)
Cumulative dosage	
Prednisone (g)	0.93 (0.16, 9)
CTX (g)	0 (0, 0.2)
HCQ (g)	1 (0, 11.6)
SLEDAI	11 (6, 17.75)
HAMD	9 (6, 13)
HAMA	7 (4, 11)

	*n* (%)

Female	153 (86.9%)
Autoantibodies	
Antinuclear antibody (ANA)	176 (100.0%)
Anti-P0 antibody	68 (38.6%)
Anti-dsDNA antibody	92 (52.3%)
Anti-Sm antibody	100 (56.8%)
Anti-U1-RNP antibody	61 (34.7%)
Anti-SSA-52 kD antibody	79 (44.9%)
Anti-SSA-60 kD antibody	112 (63.6%)
Anti-SSB antibody	47 (26.7%)
Anti-histones antibody	96 (54.5%)
Anti-nucleosome antibody	86 (48.9%)
Anticardiolipin (aCL) antibody	35 (19.9%)
Lupus anticoagulant complex (LAC)	60 (34.1%)
Active disease activity (SLEDAI > 9)	95 (54.0%)
Arthritis	58 (33.0%)
Myositis	13 (7.4%)
Urinary casts	5 (2.8%)
Hematuria	56 (31.8%)
Proteinuria	59 (33.5%)
Pyuria	65 (36.9%)
New rash	57 (32.4%)
Alopecia	41 (23.3%)
Mucosal ulcers	16 (9.1%)
Pleurisy	21 (11.9%)
Pericarditis	17 (9.7%)
Low complement	143 (81.3%)
Fever	38 (21.6%)
Thrombocytopenia	30 (17.0%)
Leukopenia	49 (27.8%)
Lupus headache	6 (3.4%)
Vasculitis	10 (5.7%)
Visual disturbance	2 (1.1%)
Seizure, psychosis, organic brain syndrome, cranial nerve disorder, and cerebrovascular accidents	0 (0%)
Depression	14 (8.0%)
Anxiety	21 (11.9%)

SLEDAI: Systemic Lupus Erythematosus Disease Activity Index; CTX: cyclophosphamide; HCQ: hydroxychloroquine; HAMD: Hamilton Depression Rating Scale; HAMA: Hamilton Anxiety Rating Scale.

**(a):** 

Independent variable	OR	95% CI	*p*
Pyuria	6.275	1.059–37.199	0.043
Hematuria	5.155	1.020–26.047	0.047
HAMA score	43.611	7.148–266.095	0.000

**(b):** 

Independent variable	OR	95% CI	*p*
Alopecia	5.460	1.358–21.949	0.017
HAMD score	30.458	6.040–153.594	0.000
Age	4.451	0.916–21.632	0.064
